# Stem Cell-Released Microvesicles and Exosomes as Novel Biomarkers and Treatments of Diseases

**DOI:** 10.1155/2016/2417268

**Published:** 2016-08-08

**Authors:** Yanfang Chen, Yaoliang Tang, Weiwen Long, Chunxiang Zhang

**Affiliations:** ^1^Department of Pharmacology & Toxicology, Boonshoft School of Medicine, Wright State University, Dayton, OH, USA; ^2^Vascular Biology Center, Department of Medicine, Medical College of Georgia, Augusta University, Augusta, GA, USA; ^3^Department of Biochemistry & Molecular Biology, BSOM, Wright State University, Dayton, OH, USA; ^4^Department of Pharmacology, Rush University, Chicago, IL, USA

Extracellular microvesicles (MVs) and exosomes (EXs) can mark the changes of their origin cells. They can transfer their carried messages (proteins, mRNAs, and microRNAs) to target cells to modulate the functions of the recipient cells. Therefore, MVs and EXs are recognized as potential biomarkers and important therapeutic targets or tools for a wide spectrum of diseases including vascular and neurological diseases and cancers [1–4]. While research in stem cells has achieved significant advances during the past decades, there is less information regarding the roles of MVs and EXs released from stem cells. The current special issue includes the selected reviews and original articles focusing on novel studies of stem cell-released MVs and EXs as biomarkers and treatments of cardiovascular and neurological diseases and cancers.

There is an urgent need to identify sensitive and specific methods for the isolation and identification of MVs and EXs, which is very important and challenging. J. Wang et al. [5] have developed novel methods for analysis of exosomes released from endothelial cells (ECs) and endothelial progenitor cells (EPCs) by combining microbeads and fluorescence quantum dots (Q-dots®) techniques. The authors showed that anti-CD105/anti-CD144 and anti-CD34/anti-KDR had the highest sensitivity and specificity for isolating and detecting EC-EXs and EPC-EXs, respectively. A. Fricke et al. [6] evaluated whether MVs shed by synovial sarcoma cells carry the tumor-specific fusion gene SYT-SSX transcripts and revealed that these MVs might serve as a diagnostic biomarker for synovial sarcoma. They also noted that more sensitive assays are needed to detect cancer specific microvesicles in the peripheral blood of cancer patients. Leukemia stem cells (LSCs) are able to self-renew, proliferate, and differentiate extensively, which are crucial for the initiation and maintenance of acute myeloid leukemia (AML). AML is an aggressive disease characterized by rapid proliferation of immature myeloid cells. Y. Wang et al. [7] reported that LSCs-released MVs support AML cell malignance and targeting of miR34a could offer a new approach for the management of AML.

Exosome-based therapy could overcome many limitations of cell-based strategy in safety, potency, efficacy, and scalability of stem cell products. The article by J. Burke et al. [8] reviewed the existing literature to highlight the current advances of stem cell-derived exosomes with particular attention to regenerative medicine in orthopaedics. A. Luarte et al. [9] provided a review on the potential therapies of stem cell-derived exosomes in CNS diseases by focusing on the neurogenic niche. This article discusses how the modulation of the adult neurogenic niches may be a therapeutic target. F. Alcayaga-Miranda et al. [10] provided a review article which discusses the current knowledge related to the angiogenic potential of exosomes of mesenchymal stem cells and methods to enhance their biological activities. H. Zhang et al. [11] performed meta-analysis based on the reports published between 2000 and 2015 to assess the clinical effectiveness of using exosomes in ischemia/reperfusion injury. They revealed that exosomes secreted from mesenchymal stem cells inhibit myocardial ischemia/reperfusion injury.

Hepatic stellate cells (HSCs), also known as liver-specific mesenchymal stem cells (MSCs), contribute to liver regeneration. R. Huang et al. [12] showed that HSC-derived MVs dose-dependently protect hepatocytes from toxicant-induced injury. Although the molecular mechanisms have not been elucidated, the beneficial effects of MVs are likely through maintaining the proliferative activity and the antiapoptotic and antiautophagic abilities of hepatocytes. B. Dai et al. [13] investigated the effects of biomaterial multilayer membranes of hyaluronic acid (HA) or chondroitin sulfate (CS) and Collagen I (Col I) on the functions and MVs release of EPCs. They found that multilayer composite membranes composed of HA/CS and Collagen I composed multilayer composite membranes can promote EPCs functions and release of miR-126 rich EPCs-MVs, which provides a novel strategy for tissue repair treatment. Therefore, combined use of biomaterials and stem cells may present a novel approach for promoting wound healing and tissue regeneration. However, further investigations in animal models and patients are required.

Stem cell-derived exosomes and MVs could be used as biomarkers of various diseases, as well as novel approaches to combat diseases. However, further experimentation must be done and more discoveries must be made in order for them to become available for clinical use. We hope that the original research articles and review articles presented in this special issue represent the current advances in this field, which will stimulate further in-depth exploration.

*Yanfang Chen* *Yanfang Chen* *Yaoliang Tang* *Yaoliang Tang* *Weiwen Long* *Weiwen Long* *Chunxiang Zhang* *Chunxiang Zhang*
